# NTRK Gene Expression Analysis in Oral Squamous Cell Carcinoma Mexican Population

**DOI:** 10.3390/dj12100327

**Published:** 2024-10-14

**Authors:** Lilibeth Stephania Escoto-Vasquez, Javier Portilla-Robertson, Josué Orlando Ramírez-Jarquín, Luis Fernando Jacinto-Alemán, Alejandro Alonso-Moctezuma, Carla Monserrat Ramírez-Martínez, Osmar Alejandro Chanes-Cuevas, Fabiola Salgado-Chavarria

**Affiliations:** 1 Oral Medicine and Pathology Department, Postgraduate and Research Division, Dentistry School, National Autonomous University of Mexico, Mexico City 04510, Mexico; lilibeth_es15@comunidad.unam.mx (L.S.E.-V.);; 2 Neurosciences Division, Cellular Physiology Institute, National Autonomous University of Mexico, Mexico City 04510, Mexico; 3 Oral and Maxillofacial Surgery Department, Postgraduate Division, Dental School, National Autonomous University of Mexico, Mexico City 04510, Mexico; 4 Dental Biomaterials Laboratory, Postgraduate Division, Dental School, National Autonomous University of Mexico, Mexico City 04510, Mexico

**Keywords:** NTRK, immunohistochemistry, pan-TRK, RT-qPCR, correlation expression

## Abstract

Oral cancer holds the sixth position in malignancies worldwide; 90% correspond to oral squamous cell carcinoma (OSCC). Diverse reports suggest that NTRK genes and their receptors are key oncogenesis regulators to tumor progression in human cancers. **Objective:** To analyze the NTRK and Trk expression and their association with clinicopathological features of OSCC in Mexican patients’ samples. **Material and Methods:** We analyzed 95 OSCC cases of pan-trk immunoexpression through a software-assisted method. Gene expression was analyzed by RT-qPCR employing the ΔΔCT method. Kruskal–Wallis and Spearman’s correlation tests were performed. **Results:** Our mean age was 62.4 (±16.9) years. A total of 37 cases were tumors in the lateral border of the tongue. Age was significantly associated with the anatomical site. 42% (40 of 95) cases were pan-trk positive. A total of 21 cases showed intense immunoexpression predominantly in poorly differentiated OSCC, with a significant correlation between immunoexpression and age and gender. Gene expression showed that poorly differentiated cases exhibited higher NTRK2 expression, while well-differentiated cases demonstrated NTRK3 significantly higher expression. **Conclusions:** Our results suggest that NTRK family expression is present in OSCC, with differential expression related to differentiation degree. Additional information about their activation or mutational status could reinforce their potential as a possible primary or adjuvant treatment target.

## 1. Introduction

Cancer represents an important worldwide health problem. In 2022, according to the GLOBOCAN, 19,292,789 new cancer cases and 9,958,133 related deaths were reported [1]. It is estimated that it is the first or second cause of death before the age of 70 in at least 112 countries, including Mexico [2,3].

Oral cancer is the sixth most common cancer worldwide. In 2022, 389,846 new cases and 188,438 deaths were reported globally, and 90% of oral cancer cases correspond to oral squamous cell carcinoma (OSCC) [4,5]. Oral cancer incidence has a marked geographical variation, higher in South Asia and European countries such as France and Hungary [6]. In Latin America, according to International Agency for Research on Cancer (IARC) data, Brazil occupies 16th place, followed by Venezuela in the 19th place, and Mexico in the 21st place [3,7,8]. Particularly for Mexico, the ASR (age-standardized rate) in lip and oral cavity cancer represents 1.2 per 100,000 incidence and 0.43 per 100,000 mortality [3]. These global and regional data reinforce that prevention strategies, early detection, adequate treatment, and pathogenesis research are needed.

OSCC mainly affects men between the sixth and seventh decade of life [9]. Clinically, it can be observed as an ulcer with irregular edges or as an exophytic growth with raised and indurated edges. Early-stage lesions are usually asymptomatic; however, as they progress, they can cause pain and inflammation [10]. OSCC can be preceded by oral potentially malignant disorders (OPMD), which are defined as clinical conditions that carry a risk of progressing to oral cancer. These OPMDs may manifest as visible precursor lesions, such as leukoplakia, erythroplakia, oral submucous fibrosis, lichen planus, oral lichenoid lesions, and proliferative verrucous leukoplakia, and often show histological signs of epithelial dysplasia [11,12]. A systematic review by Iocca et al. found that the overall malignant transformation rate is 7.9%, with varying proportions for each type of lesion [13]. Comprehensive oral examination is essential because OSCC can affect any oral mucosa area, but the tongue lateral side is the most affected place, with approximately 50% of cases, followed by the floor of the mouth, soft palate, gum, buccal mucosa, and soft palate [14,15,16].

The main risk factors associated still are tobacco and alcohol consumption, with their synergistic effect [16,17]. Other important risk factors to consider are betel quid or areca nuts related to certain demographic areas, viral infections such as HPV, poor oral hygiene, ultraviolet radiation exposure, immunological defects, genetic predisposition, and nutritional deficiency, particularly consumption of ultra-processed foods [18,19,20,21,22].

Their OSCC pathogenesis includes genetic and epigenetic aberration accumulation, with multiple molecular events that affect and modify different signaling pathways related to cell proliferation, differentiation, cell survival, and others. Identification of these deregulation participants is a way to understand the neoplasm’s biological behavior [23,24].

Trks are transmembrane receptor tyrosine kinases in the TrkA, TrkB, and TrkC families, which are encoded by NTRK1, NTRK2, and NTRK3 genes, respectively. Each is localized on a different chromosome (1q23.1, 9q21.33, and 15q25.3). Every receptor consists of an extracellular ligand-binding domain, a transmembrane region, and an intracellular kinase domain, whose phosphorylation activates components of the MAPK pathway, resulting in cell differentiation [25]. Their constitutive activation and overexpression have been associated with carcinogenesis; relatively, in all cases, these features result in a ligand-independent dimerization, phosphorylation, and downstream activation that could favor tumor progression [26,27]. In OSCC, NTRK expression has been associated with multiple biological processes, such as lymphovascular and perineural invasion, apoptosis resistance, lymph node metastasis, and worse prognosis [28,29,30].

Our aim was to analyze the NTRK and Trk expression and their association with clinicopathological features of OSCC in Mexican patients’ samples.

## 2. Materials and Methods

### 2.1. Sample Selection

This study was approved by the Institutional Research and Ethic Committee (CIE/1723/03/2024). Samples were retrieved from the histopathological paraffin block archives of the Oral Medicine and Pathology Department, Postgraduate Division of Dentistry School (ISO-9001:2015 certified CMX-C-SGC-299-2024). This study was conducted following the integral privacy notice for patients from the Dentistry School, protecting their identity. Archives from 1999 to 2022 were consulted; only complete cases with their clinical-demographic data and enough paraffin-embedded tissue, confirmed by two oral pathologists, and OSCC diagnosis were included.

### 2.2. Histochemical Analysis

Confirmed the OSCC diagnosis, all resulting cases were classified as well differentiated, moderately differentiated, and poorly differentiated using the histological grading scale postulated by Pindborg in 1997 [31], which considers the degree of keratinization, number of mitoses, and cellular and nuclear pleomorphism.

### 2.3. Immunohistochemistry Analysis

An immunohistochemistry assay was performed as reported [32]. Briefly, deparaffinization and rehydration of the 4 m slide were performed conventionally in xylene and alcohol washes. Antigenic retrieval was conducted with 10 mM citrate buffer in the microwave histoSTATION at 100 °C for 5 min in the GPR/20S histomodule (KOS Millestone, Sorisole, BG, Italy). Endogenous peroxidase, nonspecific background blocking, and permeabilization were performed with 3% hydrogen peroxide, 2% albumin-PBS solution, and 1% Triton X-100/Albumin solution, respectively. The slides were incubated overnight at 4 °C with a primary antibody for Pan-Trk (EPR17341-4, ab246551, Abcam, Cambridge, UK) with a previously standardized concentration of 1:700. Negative controls were obtained by substitution of the primary antibody by PBS. Posteriorly, the immunodetection was conducted using an ImmunoDetector DAB HRP Brown Immunohistochemistry (Bio SB, BSB 0007, Goleta, CA, USA), following the manufacturer’s protocol. The slides were observed by a Leica DM750 microscope to obtain five photomicrographs at 400× magnification from each sample by using a Leica ICC50 HD camera (Leica Microsystems (Schweiz) AG, Heerbrugg, Swiss). Image J 1.54D (NIH, Bethesda, Rockville, MD, USA) software was employed to determine cell positive proportion and immunoexpression intensity. The positive cell proportion was categorized into four scores as follows: (0) 0 positive cells, (1) 1–10% of positive cells, (2) 11–50% of positive cells, and (3) more than 50% of positive cells [33]. To determine immunoexpression intensity, a linear regression was applied (x = (y − b)/m) to convert the continuous variable of optical density and place it on the hierarchical intensity scale as follows: (0) negative, (1) mild, (2) moderate, and (3) intense.

### 2.4. RNA Extraction and RT-qPCR

Briefly, 50 μm slides of each sample were employed to obtain total RNA by the Relia Prep FFPE Total RNA Miniprep System kit (Z1002, Promega, Madison, WI, USA) used according to the manufacturer’s instructions. The RNA obtained was kept in the elution tube and quantified in a Nanodrop2000 spectrophotometer (Thermo Fisher Scientific, Rochester, NY, USA). Samples with values greater than 1.7 for the 260/280 ratio were selected; 40 ng of RNA from each sample was employed to perform quantitative reverse transcription-PCR using the GoTaq 1-Step RT-qPCR System kit (Promega, Madison, WI, USA) according to the manufacturer’s instructions in the Step OnePlus thermocycler (Applied Biosystems, Waltham, MA, USA) to 40 cycles. The primer sequences were as follows: for NTRK1 5′-TCAACAAATGTGGACGGAGA-3′ (sense), 5′-GTGGTGAACACAGGCATCAC-3′ (antisense); NTRK2 5′-ATCCATAGACACAGTATTGAC-3′ (sense), 5′-GCAGAAGCCAGATTGATT-3′ (antisense); NTRK3 5′-TGTGGCTTCTGTCTTCTT-3′ (sense), 5′-AGTTCTTCTCCTGCTTCTT-3′ (antisense); and GAPDH 5′-ACCACAGTCCATGCCATCAC-3′ (sense) and 5′-TCCACCACCCTGTTGCTGTA-3′ (antisense) as normalizing control. Relative gene quantification was calculated using the 2^-(ΔΔCt) method [32]. The experiments were performed in triplicate.

### 2.5. Statistical Analysis

For the qualitative variables, the mean and standard deviation were obtained. For quantitative variables of immunoexpression intensity and CT expression, a non-parametric Kruskal–Wallis test and Spearman correlation were performed. A *p*-value of <0.05 was considered significant (IBM, SPSS, version 22, IBM SPSS, Chicago, IL, USA).

## 3. Results

### 3.1. Clinical–Demographic Features

A total of 247 files corresponding to OSCC-diagnosed cases were reviewed. Posterior to exclusion criteria, only complete cases were considered, resulting in a final sample of 95 cases. Their age distribution ranged from 19 to 98 years, with a mean of 62.4 (±16.9) years. Well-differentiated OSCC had a mean age of 63.3 (±16.4) years, female gender predilection (n = 26), and greater involvement of the lateral border of the tongue (n = 20). Moderately differentiated had a mean age of 60.4 (±17.2) years, male predilection (n = 19), and more lateral border of tongue predilection (n = 15). Poorly differentiated OSCC had a mean age of 65.2 (±19.2) years, male gender predilection (n = 8), and more gingiva involvement (n = 4; Table 1). Our correlation analysis showed a significant association between age and anatomical area (*p* = 0.017), meaning that individuals over 60 years old showed more gingiva affection, while those under 60 years showed more lateral border of tongue affection.

### 3.2. Immunohistochemical Analysis

**Figure 1:**
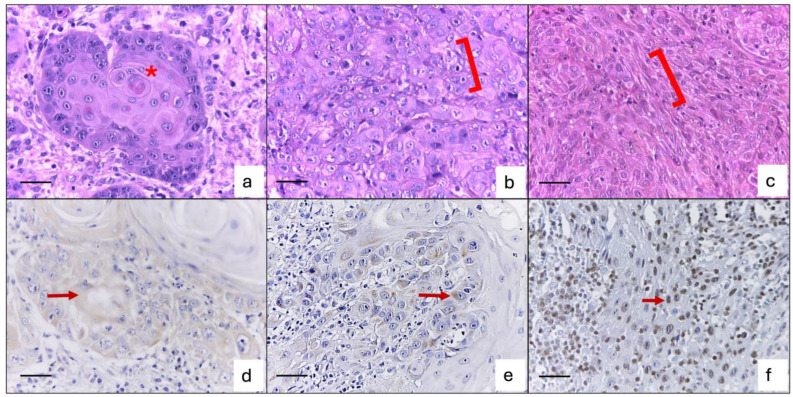
OSCC histological and immunohistochemical features. (**a**) Invasive islet of well-differentiated OSCC with cellular keratinization (asterisk), (**b**) OSCC moderately differentiated with cellular and nuclear pleomorphism (bracket), (**c**) poorly differentiated OSCC with the presence of fusiform and pleomorphic cells (bracket), (**d**) cytoplasmic mild pan-trk immunoexpression in well-differentiated OSCC (arrow), (**e**) perinuclear moderate pan-trk immunoexpression in moderate differentiated OSCC (arrow), and (**f**) strong perinuclear pan-trk immunoexpression in poorly differentiated OSCC (arrow). All photomicrographs were obtained at 400× magnification. Scale bar: 20 μm.

Immunohistochemistry assays were performed on 95 samples, identifying 40 positive cases that represent 42% of the total samples. Poorly differentiated cases showed a higher percentage of positivity. Among the 40 positive cases, 21 had a strong intensity, 32 cases exhibited a cytoplasmic expression pattern, and 24 cases showed level 1 (1–10%) of cell proportion positivity (Figure 1). The correlation analysis showed a significant association between immunoexpression with age (*p* = 0.030) and gender (*p* = 0.003), which means strong positivity was observed in men under 60 years old.

### 3.3. NTRK Gene Expression

**Figure 2:**
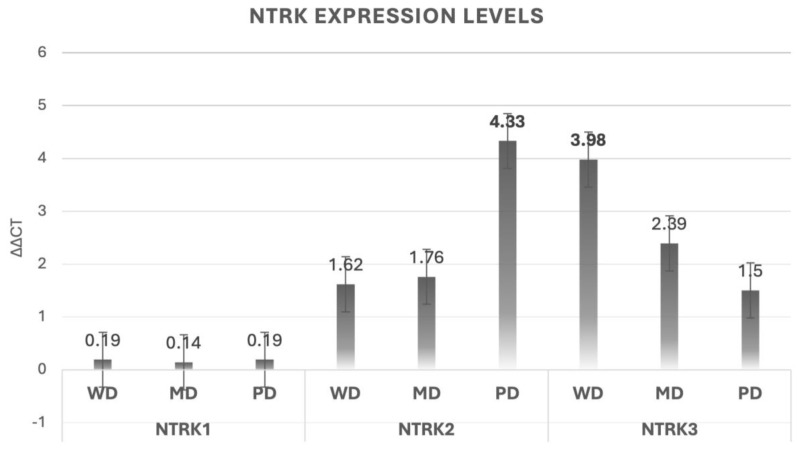
NTRK1-3 ΔΔCT results according to their differentiation degree (WD = well differentiated, MD = moderately differentiated, and PD = poorly differentiated).

RT-qPCR assays were performed on 32 samples, excluding 8 due to unsatisfactory RNA quality standards (18 samples were well differentiated, 7 were moderately differentiated, and 7 were poorly differentiated cases). Our gene expression analysis showed that NTRK1 had a minimal expression. However, for NTRK2, an important expression was observed in poorly differentiated OSCC. About the NTRK3, a significant correlation was observed with differentiation degree (*p* = 0.04), showing higher expression levels in well-differentiated OSCC (Figure 2 and Supplementary Table S1).

## 4. Discussion

Usually, OSCC occurs in elderly people, with an incidence peak between the sixth and seventh decades of life; nevertheless, there are reports that OSCC cases occur in patients between 40 and 45 years of age [9,34]. In our present study, the average age was 62 years, which is consistent with previous studies; our youngest patient was 19 years old, and 16% were under 50 years old. OSCC young patients have attracted attention because the classically associated risk factors are not reported, which could mean a different biological behavior, which could mean a harder or stricter follow-up [35].

The lateral border of the tongue has the highest incidence of OSCC, representing approximately 50% of all cases, followed by the floor of the mouth, soft palate, gingiva, buccal mucosa, and hard palate [11,36,37]. However, the anatomical site frequency varies in geographic area because the lateral border of the tongue is considered the most affected area in America and Europe, while in Southeast Asia, the buccal mucosa is the most frequent area [11]. Our results, according to the report, show the lateral border of the tongue as the more frequent, followed by the gingiva and buccal mucosa. The anatomical site is related to the histological features observed because the lateral border of the tongue consists of an epithelium that recovers muscular tissues with a dense arterial and lymphatic supply, which would facilitate invasion and metastasis. Tongue, floor of mouth, and buccal mucosa are sites particularly vulnerable to carcinogens due to the thickness of the epithelium, the higher rate of tissue turnover, and the absence of keratinization, which could provide little protection against carcinogenic products [37]. The above could be the reason for the significant correlation between age and anatomical site because gingiva OSCC was observed in patients with more than 60 years, and OSCC of the tongue and oral mucosa was presented in patients under 60 years old.

Concerning gender, it has been reported a wide male-female ratio ranging from 3.1:1 to 14:1 ratio [34,35]. This phenomenon has been attributed to historical association acceptance between classical risk factor exposure and male gender role. However, in our study, a slight prevalence of the female gender was observed. This contrast could be associated with changes in the social acceptance exposure in the female population. In Mexico, the female population represents 52% of the total population, and according to the Mexican National Survey on Drug, Tobacco, and Alcohol Consumption, an increase in alcohol and tobacco consumption among women has been observed in recent years [38].

Alcohol and tobacco exposure are risk factors that induce and promote the carcinogenesis process, which means multiple molecules participate during neoplasm formation. Trk proteins play a crucial role in neuronal function during developmental and physiological processes; they were initially identified in cancer. Its expression has been reported in several tumor tissues, including OSCC, which promotes tumor proliferation and progression [28,29,30,39]. Moraes et al. reported that the TrkB protein is overexpressed in OSCC when compared to normal oral mucosa and oral leukoplakia [40]. A limitation of this study is the absence of data on clinical or pathological stages, risk factor exposure, and longitudinal follow-up for survival analysis. The TNM system and staging are essential tools for treatment planning, estimating recurrence risk, and evaluating overall survival [41]. The Oral Medicine and Pathology Department serves as a referral center where patients are evaluated and screened for cancer. Once a patient with oral cancer is identified, they are sent to a hospital institution for TNM staging, evaluation, and comprehensive treatment. Our primary goal is to ensure early and accurate diagnosis of oral cancer and facilitate patient referrals to minimize treatment delays. Research has shown that delays in starting treatment can lead to decreased survival rates and increased local recurrence [42].

In our study, 42% of OSCC samples exhibited cytoplasmic pattern immunopositivity for pan-Trk, with strong immunoexpression in 21 cases. The Trk cytoplasmic pattern immunoexpression has been associated with NTRK2-TRAF2 fusion, which could represent an important role in antiapoptotic status associated with TRAF2, IAPs (inhibitor-of-apoptosis proteins), and inhibition of caspase activation [43]. Another interesting result was the significant correlation between immunoexpression intensity with gender and age, which means a predominant intense immunoexpression in men under 60 years old. Although there are reports that gene expression could be sex-biased, this result needs more investigation [44]. The pan-Trk (EPR17341-4, ab246551) antibody was chosen for its ability to identify Trk isoforms, allowing for subsequent specific gene determination through RT-qPCR, which can then be correlated with the clinical and histological features of OSCC.

Trk and NTRK overexpression has been associated with clinicopathological features, metastasis, and lymphovascular invasion, as well as with poor prognosis of many neoplasms, including OSCC, through diverse methodological approaches [29,30,45,46,47,48]; however, this is the first time that the gene *NTRK* family was analyzed for OSCC. In our RT-qPCR, the NTRK gene expression analysis showed heterogeneous levels. NTRK1 showed lower levels of expression; however, NTRK2 and NTRK3 showed high expression. This differential expression could reflect the complexity of neoplasmic contexts and their relationship with missense mutations, gene fusion, or overregulation of transcriptional activity in NTRK genes. Stransky et al., with their TCGA RNAseq analysis, suggested the importance of NTRK2 and NTRK3 for head and neck squamous cell carcinoma, reporting novel fusion for NTRK2 [46].

Zhou et al., in their bioinformatic analysis, showed that TrkB-T1 was the most abundant alternative splicing for NTRK2 mRNA in OSCC, correlating with NFE2L2, PIK3CA, and SOX2 pathways that promote the cancer progression [49]. Concerning NTRK3, Ogura et al. demonstrated that EWSR1 with WT1 fusion promotes NTRK3 transcription and proliferative activity in desmoplastic small round cell tumors [50]. The proliferation is an important histological feature for OSCC differentiation degree, and in our analysis, a significant correlation between NTRK3 and well-differentiated OSCC was observed. Given the fusion potential of NTRK genes, it has been reported that NTRK2 fusions may be associated with deletions in the CDKN2A/B or p16 genes [51]. Notably, overexpression of p16 has been linked to the presence of HPV in oropharyngeal squamous cell carcinoma [52]. Therefore, it would be worthwhile to assess the status of p16 in OSCC cases exhibiting NTRK alterations.

In various tumors, the identification of NTRK indicates the potential use of NTRK inhibitors like Entrectinib. Entrectinib (RXDX-101-02) is a selective ATP-competitive inhibitor targeting the tyrosine kinases TrkA, TrkB, TrkC, c-ROS1, and ALK. Clinical trials have shown that Entrectinib achieves a 100% response rate in different tumor types, including non-small cell lung carcinoma, colorectal cancer, and glioneural tumors [53]. Additionally, there is an ongoing clinical trial on head and neck carcinomas that is expected to provide further data [54].

## 5. Conclusions

Our study showed that OSCC clinical pathological features related to age and the anatomical site were, according to the report, with a significant correlation, reinforcing that observation of these clinical features is important in the oral exploration for a careful oral cancer diagnosis. In the immunohistochemical analysis, 42% of the cases showed pan-Trk expression with a predominant cytoplasmic pattern and strong intensity for poorly differentiated cases. Regarding NTRK expression, we observed a predominance of NTRK2 and NTRK3 expression, with a significant correlation of NTRK3 with the differentiation degree. Both results confirm the importance of the NTRK family for OSCC and could be exploited in terms of their activation or mutational status, among other features, for a new therapeutic approach’s development with a potential NTRK inhibitor. However, more research to expand and validate these findings is needed.

## Figures and Tables

**Table 1:** Clinical–demographic features according to OSCC differentiation degree.

Feature	Well Differentiated n= 45	Moderately Differentiated n= 37	Poorly Differentiated n= 13
Gender n (%)			
Male	19 (42.2)	19 (51.4)	8 (61.5)
Female	26 (57.8)	18 (48.6)	5 (38.5)
Mean age (SD)	63.3(16.4)	60.4(17.2)	65.2 (19.2)
Anatomic localization n (%)			
Gingiva	10 (22.2)	7 (18.9)	4 (30.8)
Buccal mucosa	7 (15.6)	5 (13.5)	2 (15.4)
Hard palate	1 (2.2)	4 (10.8)	3 (23.1)
Tongue dorsum	6 (13.3)	3 (8.1)	2 (15.4)
Lateral border of the tongue	20 (44.4)	15 (40.5)	2 (15.4)
Floor of mouth	1 (2.2)	3 (8.1)	-

The differentiation degree was determined by Pindborg postulated, considering the degree of keratinization, number of mitoses, and cellular and nuclear pleomorphism [31].

## Data Availability

The data presented in this study are available upon request from the corresponding author.

## Supplementary Materials

The following supporting information can be downloaded at: https://www.mdpi.com/article/10.3390/dj12100327/s1, Table S1: ΔΔCT expression level in OSCC samples.
