# Supplementary material for: NTRK Gene Expression Analysis in Oral Squamous Cell Carcinoma Mexican Population
Source: Dent J (Basel). 2024 Oct 14;12(10):327. doi: 10.3390/dj12100327 (PMC11506341; doi:10.3390/dj12100327)
Supplement: Supplementary file 1 [file dentistry-12-00327-s001.zip › dentistry-3165482-supplementary.pdf]

Table S1. ΔΔCT expression level in OSCC samples

Differentiation degre	Age	Gedner	Anatomical site	NTRK1	NTRK2	NTRK3
WD	85	F	G	0.74	0.87	6.54
WD	64	F	JM	0.27	0.43	7.70
WD	64	F	BLT	0.19	0.66	5.63
WD	59	M	BLT	0.32	0.65	1.23
WD	53	M	JM	0.05	2.64	2.36
WD	50	M	BLT	0.05	0.73	7.51
WD	62	F	BLT	0.06	3.77	3.40
WD	75	F	TD	0.08	2.89	8.11
WD	78	F	BLT	0.26	0.26	3.35
WD	58	F	BLT	0.16	0.35	5.33
WD	52	M	JM	0.27	1.48	1.03
WD	72	M	TD	0.24	1.34	2.31
WD	69	F	JM	0.04	3.73	1.24
WD	83	F	TD	0.03	1.66	1.19
WD	66	F	G	0.05	2.16	2.21
WD	52	F	BLT	0.07	1.92	5.74
WD	52	M	TD	0.19	2.52	1.35
WD	58	F	BLT	0.34	1.17	5.48
MD	67	F	G	0.38	1.81	1.69
MD	74	F	TD	0.06	1.07	2.92
MD	81	M	P	0.03	0.67	5.84
MD	64	F	BLT	0.03	2.95	0.29
MD	73	M	P	0.04	2.71	3.14
MD	23	M	BLT	0.09	2.07	1.56
MD	67	F	FM	0.36	1.05	1.30
PD	62	F	LM	0.17	1.27	1.80
PD	19	M	JM	0.86	10.73	0.60
PD	62	F	BLT	0.04	4.60	0.86
PD	98	M	G	0.05	1.48	0.62
PD	54	F	TD	0.03	2.49	1.17
PD	51	M	G	0.07	1.64	4.70
PD	62	M	BLT	0.10	8.10	0.74

WD: Well differentiated, MD: Moderately differentiated, PD: Poorly differentiated, F: Female, M: Male, BLT: Border lateral of tongue, JM: Jugal mucosa, TD: Tongue dorsum, P: Palate, FM: Floor of mouth, G: Gingiva and LM: Labial mucosa.
